# No Contribution of GAD-65 and IA-2 Autoantibodies around Time of Diagnosis to the Increasing Incidence of Juvenile Type 1 Diabetes: A 9-Year Nationwide Danish Study

**DOI:** 10.1155/2016/8350158

**Published:** 2016-10-13

**Authors:** Steffen U. Thorsen, Christian B. Pipper, Henrik B. Mortensen, Flemming Pociot, Jesper Johannesen, Jannet Svensson

**Affiliations:** ^1^Copenhagen Diabetes Research Center (CPH-DIRECT), Department of Paediatrics, Herlev Hospital, University of Copenhagen, Herlev Ringvej 75, 2730 Herlev, Denmark; ^2^Department of Public Health, Section of Biostatistics, University of Copenhagen, Øster Farimagsgade 5, 1710 Copenhagen K, Denmark; ^3^Department of Clinical Medicine, Faculty of Health and Medical Sciences, University of Copenhagen, Blegdamsvej 3B, 2200 Copenhagen N, Denmark

## Abstract

*Aims.* A new perspective on autoantibodies as pivotal players in the pathogenesis of type 1 diabetes (T1D) has recently emerged. Our key objective was to examine whether increased levels of autoantibodies against the *β*-cell autoantigens glutamic acid decarboxylase (isoform 65) (GADA) and insulinoma associated antigen-2A (IA-2A) mirrored the 3.4% annual increase in incidence of T1D.* Methods.* From the Danish Childhood Diabetes Register, we randomly selected 500 patients and 500 siblings for GADA and IA-2A analysis (1997 through 2005). Blood samples were taken within three months after onset. A robust log-normal regression model was used. Nine hundred children and adolescents had complete records and were included in the analysis. Cochran-Armitage test for trend was used to evaluate changes in prevalence of autoantibody positivity by period.* Results.* No significant changes in levels of GADA and IA-2A were found over our 9-year study period. No trends in autoantibody positivity—in either patients or siblings—were found. Levels of GADA and IA-2A were significantly associated with HLA risk groups and GADA with age.* Conclusion.* The prevalence of positivity and the levels of GADA and IA-2A have not changed between 1997 and 2005 in newly diagnosed patients with T1D and their siblings without T1D.

## 1. Introduction

Type 1 diabetes (T1D) is an organ-specific autoimmune disease, which has a chronic nature and serious long-term complications [1]. T1D has a strong genetic component and the cumulative incidence of T1D in monozygotic twins has been estimated to be 65%, but with latency of onset reaching decades which suggests an important role of environmental triggers and/or accelerators in the pathogenesis [2–4]. Alarmingly, many countries, including Denmark, have during the past few decades experienced an increase in incidence [5, 6]. Particularly, the rise in onset before the age of 5 years has pointed towards a more aggressive autoimmune response, for example, caused by an increase in harmful early environmental exposure [7, 8].

The destruction of *β*-cells in the islets of Langerhans is predominately caused by T-cells, but emerging knowledge also indicates a pivotal role of B-cells and autoantibodies [9, 10]. More specifically, autoantibodies form autoantigen-autoantibody complexes; the constant region of the autoantibodies—in these complexes—then binds to a specific receptor (crystallizable fragment gamma receptor (FcR*γ*)) found on antigen presenting cells (APCs), resulting in further autoantigen presentation by the APCs, hence stimulating the autoimmune response [10]. Furthermore, a more aggressive type of insulitis (high CD20+ B-cell profile) has been identified in human autopsy pancreas samples from patients with recent-onset T1D before the age of 7 years [11]. Additionally, monoclonal anti-CD20 antibodies (rituximab) directed against B-cells have been shown to partially halt *β*-cell destruction in newly diagnosed patients with T1D [12]. Whether this effect of rituximab is the cause of cross talk impairment between T- and B-cells or/and altering levels of autoantibodies remains to be proven [13]. Therefore, autoantibodies directed against specific autoantigens in the pancreas may not only serve as reliable predictors of T1D development, but may also be active players in the pathogenesis of T1D. Furthermore, levels of autoantibodies have been found to reflect the intensity of the autoimmune response in patients with T1D and a concurrent autoimmune disease [14]. However, this field of study is currently still in its cradle.

Our key objective was to examine whether increased levels of autoantibodies—as a proxy of a more aggressive autoimmune response—against the *β*-cell autoantigens glutamic acid decarboxylase (isoform 65) (GADA) and insulinoma associated antigen-2A (IA-2A) mirrored the 3.4% annual increase in incidence of T1D during our 9-year study period. Furthermore, we wanted to evaluate whether a temporal trend in autoantibody positivity existed in newly diagnosed patients with T1D and their siblings without T1D. Lastly, we wanted to elucidate/replicate whether a set of* a priori* defined covariates, that is, case status, gender, season, and HLA risk, influences the levels of GADA and IA-2A.

## 2. Materials and Methods

### 2.1. Design and Sample Population

Data for this study was derived from a population-based register of children with T1D. Initiated in 1996, the Danish Childhood Diabetes Register (*DanDiabKids*) contains information on more than 5000 newly diagnosed patients aged 0–18 years and has an associated biobank comprising blood samples from approximately 75% of all children and their first-degree relatives, of whom ~3000 are siblings below 20 years of age. A sample of 500 cases was randomly chosen where blood samples were taken less than three months after onset. The onset date was defined as the date of first insulin injection, and T1D duration thereafter was measured in months. A sample of 500 siblings with similar age and sample year distribution was chosen. Blood samples from the same family were taken within one month for 90% of the families. Eighteen patients and 21 siblings were excluded due to insufficient material for the study. The patients and siblings chosen are not necessarily from the same families, but all the siblings included have a sibling diagnosed with T1D before the age of 18 years [15].

Four hundred and eighty-two patients were included in the study of whom 255 (52.9%) were males; of the 478 siblings, 266 (55.6%) were males. Eighteen out of 482 patients were immigrants, 13 had unreported ethnicity, and the remaining were of Danish origin. The ethnicity of siblings is not reported, but since only siblings with the same father and mother as the patients are included, the distribution of ethnicity is the same in the sibling group. The sample included 203 complete sibships with at least one patient and one nonpatient, comprising 434 children in total. The sibships were used to analyse the difference between patients and siblings.

Serum samples have been stored at −80°C/−112°F after sampling and until usage.

The study was approved by the Danish Ethical Committee H-KA-20070009. All procedures followed were in accordance with the ethical standards of the responsible committee on human experimentation (institutional and national) and with the Helsinki Declaration of 1975, as revised in 2008.

Informed consent was obtained from all patients or their parents or guardians for being included in the study.

### 2.2. Analysis of GADA and IA-2A

GADA and IA-2A were measured in standard radioligand binding assays [16]. Antibody-bound antigen was separated from the free labelled antigen by Protein A Sepharose® and the unbound antigen was removed by extensive washing. The 97.5% cut-off limits for GADA and IA-2A positivity were 31 and 5 U/mL, respectively. The GADA and IA-2A assays showed mean interassay coefficients of variation (CV) of 14% and intra-assays CV of 8%. Titration above the value of 500 U/mL for GADA and 250 U/mL for IA-2A was not performed.

### 2.3. HLA-DQB1 Genotyping

Time-resolved fluorometry was used for identification of HLA-DQB1 alleles. This method is described in detail elsewhere [17].

### 2.4. Statistical Analyses

The relative change (RC) in IA-2A and GADA was modeled by robust log-normal regression taking into account the fact that measurements are both right and left censored and accounting for correlation within sibling pairs. GADA measurements are left censored at 1 U/mL (lower detection limit) and right censored at 500 U/mL (upper detection limit). IA-2A measurements are left censored at 1 U/mL and right censored at 250 U/mL. To account for correlation within sibling pairs, an inference was based on a working independence generalized estimation equation (GEE) approach. For both outcomes, the following risk factors are included in the regression: case status (patient or sibling), gender, age at sampling (<5, 5–10, and 10+ (years)), season (spring (March through May), summer (June through August), autumn (September through November), and winter (December through February)), year of sampling (1997 through 1999, 2000 through 2002, and 2003 through 2005), and HLA-DQB1 genotype (low, moderate, and high risk) (categorization of genotypes is specified in Table 1). Estimated RCs in mean are accompanied by Wald 95% confidence intervals (95% CIs). The 95% CIs are calculated on a log-scale and subsequently back-transformed. *P* values correspond to Wald tests and are performed on the log-scale. Cochran-Armitage test for trend (modified version of Pearson chi-squared test) was used to evaluate changes in prevalence of antibody positivity (GADA, IA-2A, or both), in both patients and siblings, over the three defined 3-year periods. Furthermore, the latter test was also used to evaluate changes in HLA risk grouping, in both patients and siblings.

Further, we conducted two kinds of sensitivity analyses, because we wanted to see whether this changed the effect of period on GADA and IA-2A levels in the adjusted robust log-normal regression model—as mentioned above. First, we stratified on HLA-DQB1*∗*02 and then HLA-DQB1*∗*302. In addition, we also wanted to examine whether the effect of period on GADA and IA-2A levels was independent of positivity of the other autoantibody, so a binary GADA and IA-2A “positivity variable” was constructed and included in the models. Second, we stratified individuals on whether they were above or below the median age.

All *P* values are evaluated at a 5% significance level. Due to the explanatory nature of this study, no correction for multiple testing was performed.

Analyses are made in R version 3.2.0 (https://www.r-project.org/) using the function survreg in the survival package [18].

## 3. Results

Complete measurements of GADA and IA-2A were available for 960 children. Complete records of GADA, IA-2A, and HLA-DQB1 genotype were available for 900.

Of the 960 children, 71.4% of the patients and 5.7% of the siblings were GADA positive (>31 U/mL), and 66.8% of the patients and 2.1% of the siblings were IA-2A positive (>5 U/mL). In all, 87.6% of the patients and 6.1% of the siblings were positive for a minimum of one autoantibody. Furthermore, 50.6% of the patients and 1.7% of the siblings were positive for both autoantibodies. For the 900 children with complete records, autoantibody-status results were nearly identical to those stated above.

### 3.1. Patients and Siblings

Significant higher levels of GADA and IA-2A were observed in patients compared to siblings: GADA (RC (95% CI) = 17.71 (14.18; 22.12), *P* < 0.0001) and IA-2A (RC (95% CI) = 2.20*∗*10^8^ (8.29*∗*10^6^; 5.83*∗*10^9^), *P* < 0.0001).

### 3.2. Period

**Figure 1:**
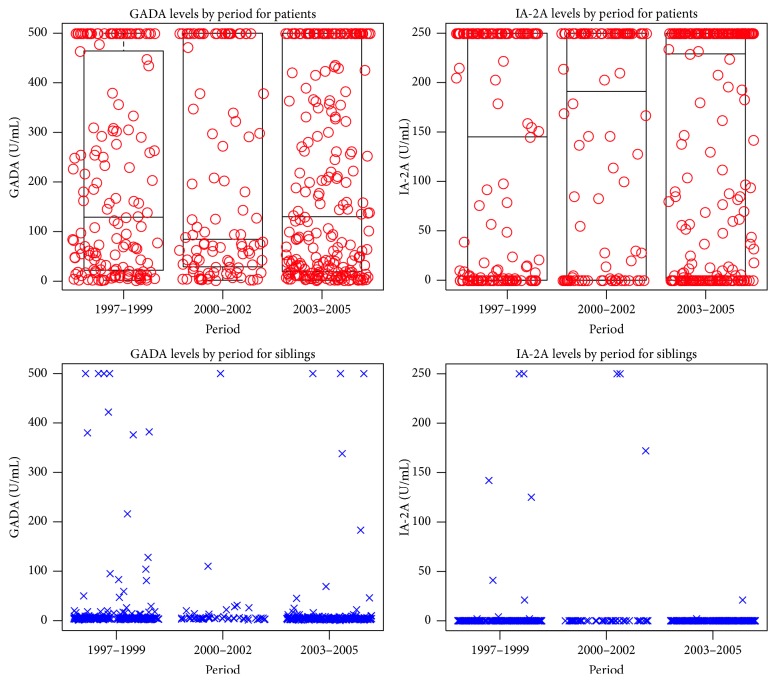
Combined box and scatter plots for GADA and IA-2A levels by period and stratification by patient status. Schematic box plots are only used in the patient stratum, due to no visual graphical gain when used in the sibling stratum, due to low levels of the two autoantibodies.

We did not observe statistical significant differences in GADA or IA-2A levels over the three periods in the adjusted model (Table 2 and Figure 1).

We also performed tests for trend using antibody status and period, stratified by case status, and none of these tests were statistically significant (data not shown) (antibody status by period is seen in Table 1).

### 3.3. Age and Sex

GADA levels were significantly higher in the older age groups when compared to the youngest group: 5–10 yrs (RC (95% CI) = 1.62 (1.01; 2.59), *P* = 0.045) and 10+ yrs (RC (95% CI) = 1.92 (1.22; 3.02), *P* = 0.005). No association was found with levels of IA-2A (Table 2). We found no differences in levels between girls and boys.

### 3.4. HLA Risk

A low risk resulted in significantly lower levels of GADA (RC (95% CI) = 0.72 (0.58; 0.90), *P* = 0.004). IA-2A showed a similar pattern for both low risk (RC = 0.030 (0.004; 0.21), *P* = 0.0005) and moderate risk (RC = 0.014 (0.001; 0.15), *P* = 0.0005) (Table 2).

We also performed tests for trend using HLA risk grouping and period, stratified by case status, and none of these tests were statistically significant (data not shown) (HLA risk groups by period are seen in Table 1).

### 3.5. Sensitivity Analyses

Two separate analyses restricted to individuals carrying (1) HLA-DQB1*∗*02 and (2) HLA-DQB1*∗*302 alleles did not show any statistical significant differences in GADA or IA-2A levels over the three periods in the adjusted model (data not shown). Furthermore, stratifying all individuals into above or below the median age did not result in any statistical significant differences in GADA or IA-2A levels over the three periods either (data not shown).

The remaining covariates were not found to be associated with GADA and/or IA-2A levels (Table 2).

## 4. Discussion

First and foremost, our results show no sign of changes in levels of GADA and IA-2A between 1997 and 2005 in patients with newly diagnosed T1D and their siblings without T1D. In addition, we did not observe any statistical significant trends of autoantibody positivity or HLA risk grouping during our study period. Secondly, we found that levels of GADA and IA-2A are associated with HLA risk; hence, newly diagnosed patients with a lower HLA risk have a weaker humoral autoimmune response against their remaining *β*-cells.

Our study is partly in line with the findings by Long et al. This study found no proof of an increase in GADA positivity that concurred with the increasing incidence of T1D in the UK (1985–2002), but the study found a temporal increase in IA-2A positivity [19]. The discrepancy in IA-2A findings between our studies may have two explanations: (1) ethnicity (hence, differences in the genetic make-up, that is, non-HLA risk loci, have been associated with IA-2A positivity) and (2) differences in statistical approach. Long et al. had a longer study period, 18 years, which rendered a higher possibility of detecting small temporal changes. The analytical approach used by Long et al. though does not use information on the actual autoantibody values above detection only whether they are positive or negative. As a consequence, less information can be extracted with their analytical approach [20–22].* In vitro* studies have proven that higher levels of GADA could enhance autoreactive T-cell responses and thereby initiate and/or fuel an autoimmune response [23]. We cannot rule out the notion that this is also the case* in vivo*, but no temporal changes in GADA levels were observed in the present study or prior mentioned study [19]. Interestingly, emerging evidence indicates that anti-idiotypic antibodies (anti-Id) can neutralize autoantibodies and downregulate their production. Anti-Id binds to the variable region (idiotype) on autoantibodies and hereby forms immune complexes, which are not detected by standard radioimmunoassays [24, 25]. We have not examined levels of anti-Id, but since we find no change in levels of GADA or IA-2A it seems that two scenarios could be possible; that is, levels of anti-Id have not changed during our study period or increased levels of anti-Id mask increased levels of GADA and IA-2A. However, it has to be mentioned that the existence and/or role of anti-Id are still debated [26].

Presentation of T1D shows a bimodal pattern with peaks at 5–7 years of age and near puberty [1]. We find a positive association between GADA levels and age, which may suggest that GADA plays a more important role in the pathogenesis of T1D in adolescents and hence may reflect different disease mechanisms. But this result could also be a result of a larger *β*-cell mass and hence stronger stimulation of the autoimmune response, but interestingly we do not find an age effect for IA-2A.

We also found that high-risk HLA-DQB1 alleles are associated with increased levels of both GADA and IA-2A in the adjusted model. It seems plausible that altered binding affinities of processed *β*-cell autoantigens presented by the HLA-DQ molecule—due to differences in the genetic make-up—result in both qualitative and quantitative differences in autoantibody production, which have also been found previously [20, 21, 27, 28].

In short, our study benefits from multiple strengths. Our study is relatively large, and it is population-based and patients are thoroughly validated. Our results are limited by only having GADA and IA-2A measurements at one time point—it would of course have been preferable if multiple measurements existed during the prediabetic phase. Furthermore, zinc transporter autoantibodies (ZnT8A) and insulin autoantibodies (IAA) were not measured and we were therefore not able to evaluate whether positivity for >2 autoantibodies showed temporal changes, but we measured two of the most common autoantibodies in T1D [28].

In sum, the prevalence of positivity and the levels of GADA and IA-2A have not changed between 1997 and 2005 in newly diagnosed patients with T1D and their siblings without T1D. Furthermore, we found no sign of genetic drift in HLA-DQB1 genotypes during the same period. Our data indicates that GADA may be more important in T1D pathogenesis above the age of 5 years.

## Figures and Tables

**Table 1:** Descriptive characteristics of the study population.

Variables	Entire study period		Period 1		Period 2		Period 3	
	(1997–2005)		(1997–1999)		(2000–2002)		(2003–2005)	
	Patient	Sibling	Patient	Sibling	Patient	Sibling	Patient	Sibling
	(*n* = 482)	(*n* = 478)	(*n* = 149)	(*n* = 217)	(*n* = 100)	(*n* = 50)	(*n* = 233)	(*n* = 211)
*Variables of main interest*								
GADA								
Median/Q1–Q3, U/mL	114/22–500	4/3–6	129/22–464	5/3–6	85/29–500	5/4–6	130/19–500	4/3–5
IA-2A								
Median/Q1–Q3, U/mL	188/0–250	0/0-0	145/0–250	0/0-0	191/0–250	0/0-0	229/0–250	0/0-0
*Autoantibody status*								
GADA pos. (>31 U/mL), *n*/%	344/71.4	27/5.7	107/71.8	17/7.8	73/73.0	2/4.0	164/70.4	8/3.8
IA-2A pos. (>5 U/mL), *n*/%	322/66.8	10/2.1	93/62.4	6/2.8	67/67.0	3/6.0	162/69.5	1/0.5
GADA & IA-2A pos., *n*/%	244/50.6	8/1.7	71/47.7	6/2.8	52/52.0	1/2.0	121/51.9	1/0.5
*Basic characteristics*								
Gender								
Female, *n*/% of total	227/47.1	212/44.4	74/49.7	99/45.6	46/46.0	18/36.0	107/45.9	95/45.2
Age at blood sampling								
Median/Q1–Q3, years	10.4/7.4–12.5	10.3/7.8–12.8	10.8/7.2–12.7	10.6/7.9–12.9	9.9/7.1–12.8	9.8/7.6–12.2	10.3/7.5–12.4	10.2/7.6–12.8
Season when blood was sampled, *n*/% from total								
Winter	121/25.1	124/26.0	38/25.5	62/28.6	31/31.0	17/34.0	52/22.3	46/21.4
Spring	123/25.5	123/25.8	38/25.5	54/24.9	25/25.0	12/24.0	60/25.8	57/27.1
Summer	109/22.6	133/27.9	30/20.1	57/26.3	24/24.0	12/24.0	55/23.6	64/30.5
Autumn	129/26.8	97/20.3	43/28.9	46/20.3	20/20.0	9.18.0	66/28.3	44/21.0

	*n* = 445	*n* = 455	*n* = 133	*n* = 211	*n* = 95	*n* = 48	*n* = 217	*n* = 196

*HLA risk groups*								
High^a^, *n*/% of total	312/70.1	228/50.1	84/63.2	112/53.0	69/72.6	20/41.7	159/73.3	96/49.0
Moderate^b^, *n*/% of total	66/14.8	43/9.5	30/22.6	19/9.0	11/11.6	7/14.6	25/11.5	17/8.7
Low/protective^c^, *n*/% of total	67/15.1	184/40.4	19/14.3	80/37.9	15/15.8	21/43.8	33/15.2	83/42.4

HLA-DQB1 genotypes are collapsed into risk categories, which are seen below.

^a^HLA-DQB1 ^*∗*^allele_1/^*∗*^allele_2: 03 : 02/99 : 99, 03 : 02/02, 06 : 04/03 : 02.

^b^HLA-DQB1 ^*∗*^allele_1/^*∗*^allele_2: 03 : 01/02, 06 : 03/03 : 02, 02/99 : 99, 06 : 04/02, 06 : 04/99 : 99, 03 : 01/03 : 02. 06 : 04/03 : 04.

^c^HLA-DQB1 ^*∗*^allele_1/^*∗*^allele_2: 06 : 02/03 : 02, 06 : 02/02, 06 : 03/99 : 99, 03 : 01/99 : 99, 06 : 02/03 : 01, 06 : 03/03 : 01, 06 : 04/03 : 01, 06 : 03/02, 03 : 04/99 : 99, 03 : 04/02, 06 : 02/03 : 04, 99 : 99/99 : 99.

99 : 99 = remaining alleles.

**Table 2:** Estimated relative differences in autoantibody levels according to the covariates in the adjusted model.

Outcome	Variable	Level	RC (95% CI)	*P* value
GADA	Status	Patient	**17.71 **(14.18; 22.12)^*∗*^	**<0.0001**
		Sibling	1	
	Gender	Females	1.12 (0.91; 1.38)	0.29
		Male	1	
	Age	10+ yrs	**1.92 (1.22; 3.02)**	**0.005**
		5–10 yrs	**1.62 (1.01; 2.59)**	**0.045**
		0–5 yrs	1	
	Period	2003–2005	0.87 (0.70; 1.09)	0.23
		2000–2002	1.04 (0.76; 1.42)	0.82
		1997–1999	1	
	Season	Spring	1.12 (0.85; 1.48)	0.41
		Summer	0.91 (0.69; 1.21)	0.53
		Autumn	0.89 (0.66; 1.22)	0.47
		Winter	1	
	HLA risk	Low	**0.72 (0.58; 0.90) **	**0.004**
		Moderate	1.39 (0.96; 2.01)	0.083
		High	1	

IA-2A	Status	Patient	2.20**∗**10^8^ (8.29**∗**10^6^; 5.83**∗**10^9^)	**<0.0001**
		Sibling	1	
	Gender	Females	0.24 (0.06; 1.07)	0.06
		Male	1	
	Age	10+ yrs	3.43 (0.24; 48.11)	0.36
		5–10 yrs	6.39 (0.42; 97.17)	0.18
		0–5 yrs	1	
	Period	2003–2005	0.68 (0.13; 3.56)	0.97
		2000–2002	1.05 (0.10; 10.72)	0.65
		1997–1999	1	
	Season	Spring	0.69 (0.09; 5.55)	0.73
		Summer	2.38 (0.26; 21.39)	0.44
		Autumn	0.34 (0.04; 2.93)	0.33
		Winter	1	
	HLA risk	Low^a^	**0.030 (0.004; 0.21)**	**0.0005**
		Moderate^b^	**0.014 (0.001; 0.15) **	**0.0005**
		High^c^	1	

^*∗*^Bold letters indicate significance at a 5% level.

HLA-DQB1 genotypes are collapsed into risk categories, which are seen below.

^a^HLA-DQB1 ^*∗*^allele_1/^*∗*^allele_2: 06 : 02/03 : 02, 06 : 02/02, 06 : 03/99 : 99, 03 : 01/99 : 99, 06 : 02/03 : 01, 06 : 03/03 : 01, 06 : 04/03 : 01, 06 : 03/02, 03 : 04/99 : 99, 03 : 04/02, 06 : 02/03 : 04, 99 : 99/99 : 99.

^b^HLA-DQB1 ^*∗*^allele_1/^*∗*^allele_2: 03 : 01/02, 06 : 03/03 : 02, 02/99 : 99, 06 : 04/02, 06 : 04/99 : 99, 03 : 01/03 : 02. 06 : 04/03 : 04.

^c^HLA-DQB1 ^*∗*^allele_1/^*∗*^allele_2: 03 : 02/99 : 99, 03 : 02/02, 06 : 04/03 : 02.

99 : 99 = remaining alleles.
